# Total Gastrointestinal Flora Transplantation in the Treatment of Leaky Gut Syndrome and Flora Loss

**DOI:** 10.7759/cureus.31071

**Published:** 2022-11-03

**Authors:** Murat Kanlioz, Ugur Ekici, Murat Ferhat Ferhatoğlu

**Affiliations:** 1 General Surgery, Flora Transplantation Instutite, Istanbul, TUR; 2 General Surgery, İstanbul Gelisim University, Istanbul, TUR; 3 General Surgery, Istanbul Okan University, Istanbul, TUR

**Keywords:** celiac disease, fecal microbiota transplantation (fmt), intestinal flora disorder, food allergy quality of life, total gastrointestinal flora transplantation, leaky gut syndrome

## Abstract

Introduction

The aim of this work was to treat patients with leaky gut syndrome (LGS) and gastrointestinal flora loss in a simple, inexpensive, permanent and effective way without the need for further treatment.

Methods

A total gastrointestinal flora transplantation (TGFT) procedure is performed by simultaneously transferring the "flora" taken from approximately 30 different anatomical sites, from the mouth to the anus, of healthy donors to the corresponding anatomical site of the patient using the endoscopic lavage method.

Results

Of the patients, 25 (44.6%) were female and 31 (55.4%) were male, totaling 56 (100%). The mean age was 32.88±15.78 years. Among the 56 patients enrolled in the study, TGFT had no efficacy in one patient, five patients underwent repeat TGFT during a mean follow-up period of 23.73±16.74 months, and the treatment was permanent in 50 patients; our success rate during the follow-up period was 89.3%.

Conclusion

In LGS, TGFT should be the gold standard treatment.

## Introduction

In various anatomical sites of our body, there exist colonizations of microorganisms, which we call flora, which we live with and host as part of a mutually beneficial relationship [1,2]. As we serve as hosts for our flora, flora members play an indispensable role in maintaining a healthy life by contributing to the physiological functions of the sites where they colonize [3-5]. The intestinal flora counts more than 10 times the total number of cells in the human body, which is recognized as a complex organ consisting of 500 to 1000 different species and 1014 bacteria [6,7]. It is crucial for human health to maintain a good symbiotic relationship between the human body and intestinal microorganisms [8]. Besides contributing to digestive functions at the site where they colonize, flora members also ensure or facilitate the performance of defense barriers and many biochemical functions [3,9]. Our gastrointestinal system (GIS) flora, extending from the mouth to the anus, contributes to the digestion and absorption of the food we eat, as well as to maintaining our endocrinological, neurological, and immunological functions in a healthy state. It is well known that digestion starts in the mouth and persists throughout the GIS [10,11].

Any structural defects and functional losses in any anatomical site/s in the GIS flora, from the mouth to the anus, may harm the functions of the preceding and subsequent anatomical sites. This is because a preceding site is the raw material supplier of the next site, a subsequent site is the natural transit route for the contents of the preceding sites, and therefore content and/or functional loss in any part of the GIS can have a chain effect on the entire system. Hence, assuming the elements of the GIS as links in a chain, it would not be wrong to say, as in the very classical example, that "a chain is as strong as the weakest link". In leaky gut syndrome (LGS), the main functional impairment occurs in the intestines, with the loss of GIS flora. In the cases of loss of GIS flora, the destroyed flora is often replaced by pathogenic microorganisms [12,13]. Given that our flora is the most important defense mechanism against pathogenic microorganisms, we can better understand how important our flora is [14]. It is common for GI flora to be lost from time to time for whatever reason, most of which recovers spontaneously without the need for any treatment. A permanent loss or decrease in our flora and its replacement by pathogenic microorganisms leads to many functional losses, resulting in numerous symptoms arising from disorders in digestion, absorption, and nutritional functions [15,16]. Although bacteria, viruses, and fungi penetrating the intestines through the diet contribute to the development of the regional intestinal immune system, they also occasionally cause serious clinical manifestations. Besides this, any disorder or abnormal colonization of intestinal flora by pathogens, including viruses and fungi, leads to the development of numerous diseases, especially diseases affecting the central nervous system, GIS, metabolism, and immune system [13,15,17]. Our flora is also vital for its role in maintaining the selective permeability of our intestines in absorption [18,19]. As a consequence of flora disorder or loss, selective permeability is impaired, which facilitates the emergence of the condition that we define in general terms as LGS (2). Whatever the cause, disruption of the composition of the GIS flora is inevitable in LGS. In LGS, the amounts of *Clostridium difficile* and *Candida albicans* increase, while the amounts of *Bifidobacterium* and *Lactobacillus* decrease [20-22]. Emerging due to a disorder in the intestinal flora, LGS has the potential to affect all systems and is considered to contribute to the development of many diseases [23]. Among the prominent treatment options for the treatment of flora disorders are the use of prebiotic-based ingredients in the diet, probiotic therapy, and fecal microbiota transplantation (FMT). FMT aims to restore intestinal flora by releasing the stool taken from a healthy donor into the intestine using endoscopic methods and thus creating healthy flora colonization [24].

Taking into consideration that the GIS flora consists of various microorganisms, that each region has a flora with a distinctive structure, that each anatomical site of the GIS has a habitat with diverse biochemical characteristics, that the GIS flora is affected by cultural, environmental, and dietary differences, that it varies according to age and gender, and as we observed after performing FMT treatments that the effectiveness of FMT treatment could not restore the entire GIS flora, we have developed a treatment method that we call "total gastrointestinal flora transplantation" (TGFT) starting from the idea of restoring the entire GIS flora at an optimum level. We identified patients with chronic GI flora loss resulting from a variety of causes [ulcerative colitis (UC), Crohn's disease (CsD), chemotherapy, radiotherapy, prolonged high-dose antibiotic treatment, prolonged cessation of oral feeding, etc.], as well as those with food allergies and malnutrition due to LGS as candidates for TGFT. We performed TGFT on patients identified as candidates for TGFT using the flora collected from a healthy GIS flora donor.

The success of TGFT is influenced by the severity and duration of the disease, the presence or absence of comorbidities, post-transplant lifestyle, donor flora quality, further treatments to be administered, and the genetic background of the patient. It is the total combination of patient and donor factors that determines the success and sustainability of flora transplantation. In cases where the factors causing flora disruption are temporary (chemotherapy, radiotherapy, antibiotic use, steroid use, cessation of oral feeding for a while, etc.), TGFT often works successfully and permanently. Yet, in diseases such as UC, CsD, lactose enteropathy (LE), and celiac disease (CD), although it contributes significantly to the remission of the diseases, the flora may deteriorate again in the following periods, depending on the episodes of the underlying disease. It is worth, at this point, putting a special parenthesis for the CD and LE. TGFT yields remarkably successful and permanent results in cases that are incorrectly defined as gluten allergy and milk allergy, but should actually be called "celiac disease-like cases" (CDLC) and "lactose enteropathy-like cases" (LELC), which are caused by eating genetically modified, industrially processed and preservative-containing foods, and which have increased almost to the extent of infectious diseases in the last 25 years, and which are incorrectly called "CD" and "LE." In most cases, CD and LE are diagnosed based on clinical findings without performing histopathologic and biochemical examinations. This leads to misdiagnosis in many cases, with the CD being diagnosed instead of CDLC and LE instead of LELC. What poses the main problem in CDLC and LELC cases is that our intestines do not recognize the products that have been genetically and compositionally modified and are not recognized by our genetic memory, and whose original structure has been disrupted by many processes during production, and which contain industrial additives, and therefore react to these products by perceiving them as harmful/foreign substances. This reaction is designated CD when it is caused by barley, wheat, and their derivatives, and LE when it is caused by milk and dairy products.

## Materials and methods

The patients with LGS-induced food allergy and malnutrition persisting for at least six months and who were found to have dysbiosis in stool microbial analysis were considered in the chronic LGS group and identified as candidates for "TGFT." Patients with missing data in the retrospective scan were not included in the study. In a setting where both the volunteer healthy GIS flora donor and the patient are present together, first, the patient and the donor are briefed about the procedure and its possible risks, and then the process is initiated after obtaining their written consent. After a general medical check-up of both the patient and the donor, examinations are performed. We seek several criteria in flora donors. The healthy volunteers without any of the exclusion criteria in Table 1 were identified as donor candidates.

**Table 1:** Exclusion criteria for donor selection in total gastrointestinal flora transplantation HBV, hepatitis B virus; HCV, hepatitis C virus; HAV, hepatitis A virus; GIS, gastrointestinal system; LGS, leaky gut syndrome.

Criteria for the last three months	Criteria for the last six months	Criteria for all times
Those hospitalized	Chemotherapy	HIV infection carrier
Live vaccine recipients	Radiotherapy	HBV infection carrier
Antibiotic users	Hormonotherapy	HCV infection carrier
Antifungal users	Immunosuppressive therapy	Chronic Infectious disease
Those who have received intravenous therapy	Intensive care treatment	Those weighing less than 20 kg/m^2^
Those who have received parasite treatment	Major surgical intervention	Those weighing more than 30 kg/m^2^
Antiviral users	Oral stop over 7 days	Diabetes mellitus
Those with a history of animal bites	Those who have had an HAV infection	Autoimmune disease
Those with open injuries	Blood transfusion	Ulcerative colitis
Those who have had diarrhea	Contact with agrochemicals	Crohn's disease
Suspicious sexual intercourse		Celiac disease
Those who have had an active infection		Lactose enteropathy
Those who gave birth		Those with GIS tumors
Those who have undergone dental treatment		Those who have undergone GIS surgery (except appendectomy)
Those who have undergone surgical intervention		Those breastfed for less than 6 months
Travel to high-risk areas		Those diagnosed with LGS
		Drug addicts
		Alcoholics
		Healthcare workers
		Prostitutes
		Homosexuals
		Those over 50 years of age
		Schizophrenics
		Autistics
		Workers in toxic industries
		Pregnant women
		Oral stop over three weeks

Donors are scored based on criteria such as breast milk intake, history of radiotherapy, chemotherapy, past illnesses, age, and habits (Table 2). Among the donors scored according to Table 2, donors with a score of 125 and above are called ideal donors, while those with a score below 70 cannot be donors.

**Table 2:** Scoring of donor quality DM, diabetes mellitus; GIS, gastrointestinal system.

Criterion	Score									
Age (years)	0-20 years → 5	21-30 years → 4		31-40 years → 3				41-50 years → 2		50+ years → 1
Breast milk (months)	1-6 months↓→ 6	6 months↑→ 9		9 months↑→ 12				12 months↑→15		24 months↑→18
Residential location	Countryside → 10		City → 3						Metropolis → 0	
Chemotherapy	Not received → 10				Received → 0					
Radiotherapy	Not received → 10				Received → 0					
Hormonotherapy	Not received → 10				Received → 0					
Antibiotics ↑ 3 weeks	Not received → 10				Received → 0					
Immunosuppressive therapy	Not received → 10				Received → 0					
Oral stop	No oral stop → 10		7 days ↓ → 5						7+ → 0	
Intensive care treatment	Not received → 10		7 days ↓ → 5						7+ → 0	
History of DM	No → 10		Prediabetes → 3						Diabetes → 0	
Allergic disease	No → 5					Yes → 0				
Autoimmune disease	No → 5					Yes → 0				
Family history of autoimmune disease	No → 2					Yes → 0				
Industrial food consumption	No → 5		Very little → 2						Yes → 0	
History of dysentery	No → 5		3≤ → 2						4≥ → 0	
History of gastroenteritis	No → 5		5≤ → 2						6≥ → 0	
GIS surgery	No → 10						Yes → 0			
Chronic medication use	No → 5						Yes → 0			
Malnutrition	No → 10						Yes → 0			
Alcohol use	No → 10		Seldom → 2						Very often → 0	
TOTAL SCORE:										
DONOR QUALITY BY SCORE										
*125 points and ↑: Ideal donor										
*100-124 points: Very good donor										
*90-99 points: Good donor										
*80-89 points: Acceptable donor										
*70-79 points: Poor quality donor										
*69-↓ points: Very poor quality donor										

If, following the examinations, tests, and consultations, there is no evidence of any impediment to general anesthesia, gastroscopy, and colonoscopy in the patient and donor, then the informed consent forms for TGFT are obtained from both the patient and the donor. The patient and the donor are required to be free of any antibiotic use for at least 21 days prior to the scheduled date of TGFT and not to have taken anticoagulant drugs in the last five days. A colonoscopy preparation diet is started on both the patient and the donor five days before the procedure. Also, both the patient and the donor are given oral bowel cleansing solution twice 12 and 16 hours before the TGFT, and they are subjected to rectal enemas twice 2 and 12 hours before the procedure, thus ensuring bowel cleansing.

The collection of flora from the donor and its transfer to the patient is performed under general anesthesia in the operating room. First, the donor is operated on, where endoscopic access is made through the mouth and the duodenum is advanced up to 30th cm. The procedure is proceeded if no active bleeding, infection, tumoral lesion, and any suspicious findings are found in the examined areas. Dividing the area from the mouth to the 30th cm of the duodenum into ca. 15 separate anatomical sites, lavage is performed from each site separately, starting from the distal duodenum and backward at intervals of ca. 5-10 cm, and the lavage content of each site aspirated is placed in separate, sealed, sterile containers and stored at 37°C (Bain-marie). Following completion of upper GI flora collection, a colonoscopy is performed up to the distal 30th cm of the ileum. Flora collection is started if no active bleeding, infection, tumoral lesion, or any suspicious finding is found in the colonoscopic examination. Dividing the area between the anus and the distal 30th cm of the ileum into 15 consecutive anatomical sites, the flora is collected by performing lavage and aspirating back through the colonoscope for each site separately. Lavage is performed sequentially starting from the distal 30th cm toward the anus. The lavage fluid aspirated from each site is placed separately in sealed and sterile containers and stored at 37°C (Bain-marie). In all lavage procedures, 50-75 cc of saline physiologic (0.9% NaCl) is used for each anatomical site. The 0.9% NaCl solution used for the lavage procedure is prepared at 37°C. Having been taken from the donor first and stored at 37°C, the flora materials are transferred to the recipient during the same session right after the procedures performed on the donor are over and while the patient is under general anesthesia. First, upper GI endoscopy is performed, progressing to the 30th cm of the duodenum. If during the examination, no active bleeding, infection, tumoral lesion, or any suspicious findings are found, the lavage materials collected from the donor and stored at 37°C are injected sequentially into the relevant sites using the endoscopy device, taking into account the same order and anatomical site from which and in which sequence they were collected from the donor. The flora cultivation procedure follows a sequence from distal to proximal (from the duodenum to the oral cavity). Then, the entire colon and ileum are observed through colonoscopy by advancing 30 cm. If during the examination, no active bleeding, infection, tumoral lesion, or any suspicious findings are found, the lavage materials collected from the donor and stored at 37°C are injected sequentially into the relevant sites using the colonoscopy device, taking into account the same order and anatomical site from which and in which sequence they were collected from the donor, and where the procedure is performed sequentially from the ileum to the anus. Once the procedure is successfully completed, the patient is left under general anesthesia for another 30 minutes, after which the procedure is terminated and the patient is awakened. On awakening, the patient should be prevented from straining too much, and too deep tracheal aspiration should be avoided.

Ideally, TGFT should be performed using flora that is enriched from healthy multiple donors. Yet, due to the limitations in finding healthy donors, the number of patients in whom we performed TGFT with multiple donors was only six in our study. In the selection of donors, the donor's genetic kinship with the patient is sought. It is also preferable that the patient and the donor are of the same gender, if possible.

This study was conducted in line with the “Helsinki Declaration” and approved by the local ethics committee.

The results were recorded and analyzed using the SPSS statistical software version 25 (IBM Corp., Armonk, NY, USA). The differences between the groups were evaluated on the basis of the Student’s t-test. A p-value of ˂0.05 was considered significant.

## Results

Of the patients, 25 (44.6%) were female and 31 (55.4%) were male, totaling 56 (100%). The mean age was 32.88±15.78 years and the median age was 33 years, with the youngest age being 5 years and the oldest age being 68 years. Of the patients, 11 (19.6%) were aged between 5 and 18 years, 38 (67.9%) were aged between 19 and 50 years, and 7 (12.5%) were aged between 51 and 68 years. The patients were distributed by body mass index (BMI) as follows: 17 (30.4%) below 20.00 kg/m^2^, 23 (41%) 20.01-25.00 kg/m^2^, 11 (19.6%) 25.01-30.00 kg/m^2^, 4 (7.2%) 30.01-40.00 kg/m^2^, and 1 (1.8%) above 40.01 kg/m^2^ (Table 3).

**Table 3:** Distribution of patients by gender, age, and BMI n, number of patients; BMI, body mass index.

Distribution by gender	Distribution by age	Distribution by BMI
Female 25 (44.6%)	Mean: 32.88±15.78 years	20.00 kg/m^2^≤ → n: 17 (30.4%)
Male 31 (55.4%)	Median: 33 years	20.01-25.00 kg/m^2 ^→ n: 23 (41%)
	Min: 5 years	25.01-30.00 kg/m^2^ → n: 11 (19.6%)
	Max: 68 years	30.01-40.00 kg/m^2^ → n: 4 (7.2%)
		40.01 kg/m^2^≥ → n: 1 (1.8%)
	5-18 years → n: 11 (19.6%)	
	19- 50 years → n: 38 (67.9%)	
	51-68 years → n: 7 (12.5%)	
TOTAL → n: 56 (100%)	TOTAL→ n: 56 (100%)	TOTAL→ n: 56 (100%)

Neither donors nor patients had any serious complications during the TGFT procedure. There were only six TGFT procedures that were postponed to another session due to insufficient bowel cleansing of the recipient or donor. The donor scoring scale revealed that the mean donor score was 102.91±8.95 and the median score was 103.5, with the lowest score being 88 and the highest 122.

The mean time elapsed from the onset of LGS symptoms until performing TGFT was 23.73±16.74 months, and the median was 28 months, with a minimum time period of 6 months and a maximum time period of 72 months. The mean follow-up period of the patients following TGFT was 35.95±34.42 months, and the median was 36 months, with a minimum follow-up period of 2 months and a maximum follow-up period of 148 months (Table 4). Out of the 56 patients who received TGFT, one patient failed to achieve any efficacy, and five patients underwent repeat TGFT at a mean follow-up period of 23.73±16.74 months. Among the 56 patients included in the study, the number of successfully completed TGFTs during the follow-up period was 50, and our success rate was 89.3%.

**Table 4:** Duration of disease before TGFT and follow-up periods after TGFT TGFT, total gastrointestinal flora transplantation.

	Disease duration before TGFT (months)	Follow-up period after TGFT (months)
Mean±SD	23.73±16.74	35.95±34.42
Median	28	36
Min.	6	2
Max.	72	148

Before the emergence of LGS symptoms, the diagnoses of the patients were as follows: one was diagnosed with UC, two with CsD, seven with CD, five with LE, six with irritable bowel syndrome (IBS), one with GI malignancy, seven with non-GI malignancy, seven with autism, and one with Behçet's disease. During the period from the emergence of LGS until the TGFT, the diagnosis of UC increased to two, CsD to three, CD to 12, LE to 15, and IBS to 22. A comparison of the pre-LGS and pre-TGFT periods revealed a significant difference in the number of comorbidities (p˂0.05). Upon evaluation eight weeks after TGFT treatment, UC was found to persist in one patient, CsD in one patient, CD in two patients, LE in one patient, and IBS in three patients. When the comorbidity rates before TGFT and eight weeks after TGFT treatment were compared, the differences between the values measured for each were found to be significant (p˂0.05). Of these diagnoses, UC and CsD were diagnosed by endoscopic examination, whereas CD and LE were diagnosed based on the clinical findings without performing any histopathologic examination (Table 5).

**Table 5:** Table of cases before LGS, during LGS, and eight weeks after TGFT LGS, leaky gut syndrome; TGFT, total gastrointestinal flora transplantation.

	Number of cases before LGS diagnosis		Number of cases after LGS diagnosis			Number of cases eight weeks after TGFT	
Ulcerative colitis	1	←p˂0.02 →		2	←p˂0.02→		1
Crohn's disease	2	← p˂0.04→		3	←p˂0.01→		1
Celiac disease	7	← p˂0.03 →		12	←p˂0.001→		2
Lactose enteropathy	5	← p˂0.01→		15	←p˂0.0001→		1
Irritable bowel syndrome	6	←p˂0.002→		22	←p˂0.0003→		3

Before the diagnosis of LGS, six patients received radiotherapy and chemotherapy together, and one patient received chemotherapy alone. A total of 22 patients had long-term systemic steroid administration, while 38 patients had a history of antibiotic use for 21 days or more, at least once in their lifetime, and 27 patients had a history of intensive industrial food consumption. A total of 14 patients had a history of autoimmune diseases (arthritis, Hashimoto's thyroiditis, psoriasis, polycystic ovary, etc.). There was a history of the need for long-term antihistamine use in 19 patients. Of the patients, 16 reported a history of intensive care treatment, and 10 reported oral stops for seven days or more. There was glucose intolerance (prediabetes) or diabetes mellitus (DM) in 14 patients, a history of allergic disease in 15 patients, and a history of chronic drug use in 27 patients. Of the patients with a history of surgical intervention, 12 had an appendectomy, three had a tonsillectomy, two had a cholecystectomy, and one had a left hemicolectomy. It was also found that breast milk intake was none in 8 (14.3%) patients, less than six months in 36 (64.2%) patients, between six and 12 months in 8 (14.3%) patients, and 12 months or more in 4 (7.2%) patients. Six patients had a family history of LGS. All of the patients had more than one comorbid factor (Table 6).

**Table 6:** Pre-LGS comorbid factors in patients LGS, leaky gut syndrome; GIS, gastrointestinal system.

	Number of patients (n)
Chemotherapy	1 (1.8%)
Chemotherapy(+)radiotherapy	6 (10.7%)
Antibiotics use, 21 days↑	38 (67.9%)
Intensive industrial food consumption	27 (48.2%)
History of autoimmune disease	14 (25%)
Immunosuppressive drug use	22 (39.3%)
Long-term antihistamine use before LGS	19 (33.9%)
Intensive care treatment	16 (28.6%)
Oral stop, seven days↑	10 (17.9%)
Glucose intolerance or diabetes mellitus	14 (25%)
History of allergies	15 (26.8%)
Chronic medication use	27 (48.2%)
Appendectomy	12 (21.4%)
Tonsillectomy	3 (5.4%)
Cholecystectomy	2 (3.6%)
Left hemicolectomy (malignancy)	1 (1.8%)
Behçet's disease	1 (1.8%)
Autism	7 (12.5%)
Never breastfed	8 (14.3%)
Non-GIS malignancy	7 (12.5%)
Family history of LGS	6 (10.7%)

The pre-TGFT period was preceded by comorbid autoimmune disease in 31 patients, glucose intolerance or DM in 26 patients, steroid use in 36 patients, and antihistamine use in 42 patients. Eight weeks after TGFT, glucose intolerance or DM was present in 14 patients, steroid use in 12 patients, and antihistamine use in eight patients (Table 7).

**Table 7:** Pre- and post-TGFT findings TGFT, total gastrointestinal flora transplantation.

	Number of patients before TGFT	Number of patients 8 weeks after TGFT
Comorbid autoimmune disease	31	Not measured
Glucose intolerance or diabetes	26	14
Steroid use	36	12
Antihistamine use	42	8

Before TGFT, 46 (79.6%) patients had a mood disorder. Of the patients, 25 had depression, three had major depression, one had bipolar affective disorder, and nine had autism and a history of aggression when consuming certain foods. The examination performed eight weeks later in patients who underwent TGFT showed that 16 (28.6%) patients still had an ongoing mood disorder, while 40 (71.4%) patients were found to be normal. Assessing the psychiatric picture, it was found that 39 (69.6%) of the patients were normal, 7 (12.5%) were depressed, 1 (1.1%) had a bipolar affective disorder in remission, whereas 9 (16.1%) patients with autistic symptoms were found to be in significant remission, whose aggressiveness disappeared, autism symptoms regressed, and who showed positive improvements in dialog with others and in learning processes. It was also found that there was no longer major depression in the patient eight weeks after TGFT. The assessment performed eight weeks after TGFT treatment showed that mood disorders in 46 (79.6%) patients regressed to 16 (28.6%) (p˂0.001) (Table 8).

**Table 8:** Pre- and post-TGFT psychiatric findings TGFT, total gastrointestinal flora transplantation.

		Before TGFT (n: number of patients)	8 Weeks after TGFT (n: number of patients)
Depression		25	7
Major depression		3	0
Bipolar affective disorder		1	1
Mood disorder	Yes	46 (79.6%)	16 (28.6%)
	No	10 (20.4%)	40 (71.4%)
	Total	56 (100%)	56 (100%)
Autism		9	Significant remission

While, in 18 (32.14%) patients, the stool zonulin test was normal before TGFT, it was elevated in 38 (67.86%) patients. The results of the stool zonulin Test were as follows: median 204.50 ng/mL, mean 289.05 ng/mL (std. dev. 271.74), min: 28 ng/mL, max: 1207 ng/mL (reference range: ˂107 ng/mL). The stool zonulin test performed eight weeks after TGFT showed that 21 patients (37.5%) had elevated levels, while 35 patients (62.5%) were within normal limits. The zonulin test results were as follows: min: 26 ng/mL, max: 624 ng/mL, median: 87.5 ng/mL, mean: 126.54 ng/mL (std. dev. 116.79). There was a statistically significant difference between the values obtained in zonulin measurements before and eight weeks after the procedure (p˂0.001) (Table 9).

**Table 9:** Pre- and post-TGFT stool zonulin test results TGFT, total gastrointestinal flora transplantation.

	Before TGFT	8 Weeks after TGFT
Those with elevated zonulin test values	n: 38 (67.86%)	n: 21 (37.5%)
Those with normal zonulin test values	n: 18 (32.14%)	n: 35 (62.5%)
Total	n: 56 (100%)	n: 56 (100%)
n: number of patients		
Mean (ng/mL)	289,05±271,74	126,54 ±116,79
	(p˂0.001)	
Median (ng/mL)	204.50	87.50
Max (ng/mL)	1207	624
Min (ng/mL)	28	26
(Reference range: 0-107 ng/mL)		

Following TGFT, donors were discharged after 8 hours. The patients who underwent flora transplantation were discharged after a minimum stay of 8 hours, a maximum stay of 192 hours, a median stay of 17 hours, and a mean stay of 23.93 hours (std. dev. 25.65). The clinical follow-up after flora transplantation did not reveal any complications in the donors. In recipients of flora, however, nine patients had a fever, three had abdominal pain, and three had a fever and abdominal pain together. These complications were relieved by simple symptomatic treatments.

The onset of the post-TGFT efficacy was between 1 and 7 days, with a median of 2 days and a mean of 2.63 days (std. dev. 1.18). Most of the TGFT symptoms disappeared between 5 and 21 days, with a median of 10 days and a mean of 10.93 days (std. dev. 3.84). The time period when the post-TFGT efficacy was maximal was between 15 and 60 days, with a median of 23 days and a mean of 24.45 days (std. dev. 9.85). However, one patient had no change in the existing clinical picture after flora transplantation (Table 10).

**Table 10:** Assessment on TGFT efficacy TGFT, total gastrointestinal flora transplantation.

	Mean±SD (days)	Number of patients (n)
Time of the onset of TGFT efficacy	2.63±1.18	56
When most of the complaints regress after TGFT	10.93±3.84	56
When the effect is at maximum after TGFT	24.45±9.86	56

Of the patients who were assessed eight weeks after TGFT, symptoms related to LGS were reported as completely disappeared in 25 patients (44.6%), significantly disappeared in 26 patients (46.4%), and partially disappeared in four patients (7.1%), with no improvement in one patient (Table 11). On asking the patients about the efficacy of the treatment, 47 patients (83.9%) rated it as very good, six patients (10.7%) as good, two patients (3.6%) as little, and one patient (1.8%) as no efficacy at all.

**Table 11:** Assessment of total gastrointestinal flora transplantation results after eight weeks

	Number of patients (n)	%
Totally disappeared	25	44.6
Significantly disappeared	26	46.4
Partially disappeared	4	7.1
No changes	1	1.8
Total	56	100

## Discussion

The outcomes we achieved with FMT, which we started to administer about 20 years ago after completing the experimental phases, encouraged us and enlightened our path. During that period, our first FMT procedures were performed in LGS cases that developed secondary to the treatment of cancer patients (surgery, chemotherapy, radiotherapy, steroids, immunosuppressive treatments, etc.). We were thrilled by the striking improvements in LGS symptoms in our FMT-treated patients, which at the same time held many unknowns for us. There have been numerous treatment experiences where we have achieved very good results in some patients, with more limited efficacy in some and no efficacy at all in others. Despite the negative results we obtained in the FMT application, which had many unknowns during that period and adversely affected our motivation, we never gave up and continued our studies. By the end of the processes in which we examined the unfavorable results, we pursued and expanded our studies oriented toward the TGFT. Admittedly, we had to perform many retries before we achieved the current performance of the TGFT. I believe that it would be better to share with you that we have such a story before starting the discussion.

Since that we have not yet found a study on TGFT in the literature, making a comparison of similar samples would not be possible. Yet, it would be possible to compare the efficacy of FMT and those of probiotic therapies as the closest treatment. In TGFT treatment, which we started to perform after attaining more than 500 patients in FMT treatment, we included 56 patients with complete parameters in our retrospective study, despite exceeding the 300-patient limit in the last 15 years. We would like to share with you our experiences in FMT treatments after performing more than 300 TGFTs. Our analysis of the data shows very striking results, and a need for larger case series is evident.

The most significant physiopathologic process in LGS starts with the loss of flora, leading to the emergence of clinical manifestations. In their study, Burger-van et al. reported the flora to be the most important component that creates the intestinal barrier and contributes to its maintenance [25].

We excluded BMI as an application criterion when performing TGFT in patients with LGS. However, as a criterion for eligibility as a donor, we made sure that the candidate was in the BMI range of 20-30 kg/m^2^. In the consensus report on "performing fecal microbiota transplantation" in 2021, Keller JJ et al. recommended that donors should be in the BMI range of 20-25 kg/m^2^ [24]. I completely agree with Keller JJ et al. that they are quite right to target more ideal donors by limiting their margin, but due to our difficulties in finding donors in TGFT, which is a complicated process, we had to keep the margin a little wider and put it in the range of 20-30 kg/m^2^. We are pleased that the donor criteria we applied based on our clinical experience are partially compatible with the international consensus report, albeit after many years. Having repeatedly observed poor flora quality in obese and underweight donors, we set a BMI of 20-30 kg/m^2^ as the optimal donor criterion. Keller JJ et al. took the donor age range as 18-60 years in the same study [24]. In our study, however, we applied an upper age limit of 50 years, without setting a lower limit for donor age. The reason why we did not set a lower limit is that we aimed to select a donor as close to the patient's age as possible for patients in our pediatric age group. Besides, the reason why we set an upper age limit of 50 years as the upper limit for donor candidates is that we consider the increase in comorbid factors with aging as an important factor that decreases the quality of flora. In their animal studies, Ahmedi et al. reported that they found a higher rate of intestinal flora disorder in old and obese mice [26]. In another study, Ruiz et al. reported a change not only in the composition of the gut microbiota with aging but also in the efficiency of the microbiota in fulfilling important functions [27]. In their study, Marchesi et al. found a gradual increase in DM and reported that the intestinal microbiota deteriorated accordingly [28]. Given that DM is increasing rapidly due to changes in dietary habits, it appears that more attention should be paid to the protection of intestinal flora. The eighth edition of the International Diabetes Federation (IDF) Diabetes Atlas reports approximately 425 million people with DM worldwide. It is underlined that DM rates increase with aging. By 2045, the number of DM patients is estimated to reach 700 million [29]. Many studies on DMs have so far revealed that the number of beneficial bacteria in the intestines decreases while the number of harmful bacteria and fungi increases [21,22]. We tried to prefer younger donors in our study whenever we had a chance to choose, but we were careful not to exceed the upper age limit of 50 years because of the rapid increase in comorbid factors, especially DM, with advancing age. It is also demonstrated many times by our clinical empirical observations that a TGFT performed by young donors tends to yield a more successful and sustainable outcome.

We also observed that the number and severity of comorbid factors and their multiple combinations affected the success of transplantation. Patients tend to accept every therapeutic development because of the disease process they are going through, and they always push us very hard to perform the transfer as soon as possible with the available donor candidates they can find for TGFT. For us, the most important, troublesome, time-consuming, and exhausting process has always been donor analysis. Nevertheless, we do not always find the ideal donor. We are aware that the most important parameter that determines the success of TGFT is finding a quality donor. At this point, DM represents the most decisive factor for both the donor and the recipient. We have always been challenged by unregulated DM. However, we observed that DM is much easier to control after TGFT. In the pre-TGFT phase, 26 patients were reported to have glucose intolerance (prediabetes) and DM. The number of patients diagnosed as glucose intolerant (prediabetes) and DM decreased to 14 eight weeks after TGFT treatment. As reported by Mishra et al. in their 10-year clinical study results, microbes living in our intestines (microbiota) contribute not only to the maintenance of normal metabolic function but also to the physiopathology of metabolic diseases such as obesity and diabetes [30]. When selecting donors, we applied the criterion of having breastfed for at least 12 months. Our analysis of the patients in whom we performed TGFT showed that only four (7.2%) of the patients had a history of breastfeeding for 12 months or more and the mean duration of breastfeeding was 4.21±3.52 months. Demir et al. reported in a study conducted in the same geographical region in Turkey that only 12.3% of mothers breastfed their children for at least 12 months (mean 7.7±3.3 months) [31]. The fact that the duration of breastfeeding in the patients in whom we performed TGFT was about half of the duration reported by Demir et al. reveals that breast milk is one of the important parameters in the formation and maintenance of intestinal flora. It is recommended by the World Health Organization that infants should be breastfed for at least 24 months [32]. In their research, Rinella et al. reported that several factors, starting with the transfer of maternal microbes during fetal development, change and reshape the human gut microbiota later on, including the type of birth, method of breastfeeding, infant weaning from milk to solid food, and interactions [10].

We consider that one of the most qualified findings in our study was the results related to breastfeeding. In many research studies, the role of breastfeeding in the formation and maintenance of flora has been emphasized [33-35].

LGS and flora loss often have underlying and mutually triggering causes. The results of our study revealed that before being diagnosed with LGS, one of our patients was diagnosed with UC, two with CsD, seven with CD, five with LE, six with IBS, one with GI malignancy, seven with non-GI malignancy, seven with autism, and one with Behçet's disease. Following the loss of flora and the emergence of LGS, the diagnosis of UC increased to two, CsD to three, LE to 15, CD to 12, and IBS to 22. Both these diseases and the treatments prescribed to treat them facilitate the development of LGS and make the process a vicious circle. Even though many of the treatments prescribed for LGS (steroids, immunosuppressives, antibiotics, etc.) seem to relieve the patient in the short term, they further exacerbate the loss of flora. As shown in our study, the numbers of UC, CsD, CD, CD, LE, and IBS increased after the diagnosis of LGS and flora loss and due to the treatments administered in the pre-TGFT period. Such diseases facilitate the loss of flora, and loss of flora facilitates the emergence of these diseases. That is why not only flora restoration but also significant remissions and cures are observed in these diseases in the aftermath of TGFT. The assessment performed eight weeks after TGFT as part of our study revealed a regression of all these diseases. In their study, Nishida et al. reported that intestinal flora disorder may be a very important factor in the etiopathogenesis of UC and CSD with IBD [36]. Another study linked changes in gut microbiota with the development and progression of IBD (especially UC and CsD) [37]. In the study by Lee et al., it was reported that intestinal flora has key functions in digestion and absorption of nutrients from the intestines, control of pathogens, regulation of immunity, development of intestinal absorption surface and barrier function, and production of antimicrobial peptides and mucosal properties including mucus and repair, and that flora disorders may predispose to IBD [37].

It was also reported in two different studies that if proper gut flora is not created, the function of the intestinal immune system may be impaired, leading to increased incidence and/or morbidity of certain intestinal diseases, including UC, CsD, and colorectal cancer [38,39].

An analysis eight weeks after TGFT treatment in our study found that one patient continued to have UC symptoms, albeit with a decrease, while the other patient had complete remission of UC symptoms. While two out of three CsDs achieved complete remission, one patient achieved partial remission. Among 12 patients diagnosed with CD, two achieved partial remission, while 10 achieved complete remission. Out of the 15 patients diagnosed with LE, one patient was in partial remission, while 14 patients achieved complete remission. Among 22 IBS patients, three patients were in partial remission and 19 patients were in complete remission. Restoration of flora with TGFT has led to significant remissions in both IBD and IBS.

Before the diagnosis of LGS, our study included 38 patients with a history of antibiotic use for three weeks or more at least once in any period of their lives, 27 patients with a history of intensive industrial food use, 22 patients with a history of immunosuppressive drug use, 19 patients with a history of long-term antihistamine use in the past, 16 patients with a history of intensive care treatment, 10 patients with a history of oral stop for seven days or more, 26 patients with glucose intolerance or DM, 15 patients with allergic diseases, and 27 patients with chronic drug use. The patients diagnosed with LGS had a combination of at least one or more of the above-mentioned factors. It is necessary to administer treatments more carefully by considering comorbid parameters and to take some protective supportive measures in patients with a background that may constitute a basis for LGS. In their study, Lui et al. reported serious digestive system problems in patients who had to be hospitalized in intensive care for COVID-19 treatment [40]. The study by Lui et al. suggests that the combination of these factors including intensive care, high doses of antibiotics, high doses of steroids, partial oral stop, and the presence of a comorbid metabolic disease may cause digestive system problems. Zhang et al. reported in their study that metabolic diseases, especially DM, were factors in the impairment of intestinal flora [41]. Yet, another study reported a decrease in intestinal permeability in diabetic patients in whom the disease was regulated with treatment [42]. It was reported in a different study that broad-spectrum antibiotics caused damage to the intestinal flora [43].

Another striking finding in our study was a much higher appendectomy rate compared to the general population. Despite the fact that the appendectomy rate in the whole population was reported to be 5.8% in Europe, this rate was 21.42% in our study [44]. It is well known that the appendix contributes to the immune system. We suggest that appendectomy is important in maintaining both intestinal immune balance and intestinal flora. Shi et al. underlined in their study that the appendix vermiformis is essential for lymphoid activation and mesenteric defense [45].

In our study, significant clinical improvements were observed in all nine patients with autism after TGFT. We observed remarkably positive improvements in patients with autism after TGFT in terms of getting in contact with the social environment, school success, and reduction of aggressive behaviors. In their study, Fingold et al. reported that the intestinal flora of children with autism was disrupted [46]. Our favorable results obtained in patients with autism with intestinal flora restoration suggest that we need to conduct more in-depth research on the subject.

Following the treatment with TGFT, the rate of mood disorder decreased from 79.6% to 28.6%. In addition to the regression of the LGS picture and the formation of healthy flora, one of the most dramatic improvements has been observed in mood disorders. There are several studies indicating that flora dysfunction and habitat disruption in the GIS play an important role in neuropsychiatric disorders such as schizophrenia, major depressive disorder, and bipolar disorder [47,48].

The parameter that is most indicative of restoration of the damage caused by the loss of LGS and flora in the gut by TGFT is the result of the stool zonulin test performed before and eight weeks after TGFT. The results of the stool zonulin test before the procedure were elevated in 38 patients (67.9%) with a mean of 289.05±271.74 ng/mL (N: 0-104 ng/mL). Eight weeks after TGFT treatment, only 21 patients (37.5%) had elevated results in the stool zonulin test and the mean of the stool zonulin test results decreased to 126.54±116.8 ng/mL after treatment. We believe that following TGFT, there are dramatic improvements in mood disorders due to the normalization of serotonin secretion from the intestines along with the normalization of the intestinal habitat and the overcoming of the zonulin-induced pressure on the central nervous system.

Recently, it has been shown that dysbiosis, which develops due to disruption of intestinal flora, is strongly associated with obesity, allergies, autoimmune disorders, IBS, IBD, and psychiatric disorders [49,50].

Limitations

Ideally, TGFT should be performed using flora that is enriched from healthy multiple donors. Yet, due to the limitations in finding healthy donors, the number of patients in whom we performed TGFT with multiple donors was only six in our study. In the selection of donors, the donor's genetic kinship with the patient is sought. It is also preferable that the patient and the donor are of the same gender, if possible. Moreover, the small sample size and the lack of a comparison group are also among the limiting factors.

## Conclusions

We believe that the most effective treatment for LGS and flora loss is the preventive measures designed taking into account the factors that facilitate the development of the disease. Despite a number of factors that trigger the loss of flora, we consider that we can prevent these diseases in a significant proportion of cases by paying more attention to the antibiotic, steroid, and immunosuppressive treatments due to their widespread use. Our clinical observations suggest that the earlier TGFT is performed after the loss of LGS and flora, the more successful results are obtained, and the longer the disease duration, the lower the success rate of TGFT. It is crucial to diversify the flora with multiple donors, if possible, to obtain better quality flora. We also need to review our previous habits to preserve the flora after TGFT.
